# Investigating Human Visual Sensitivity to Binocular Motion-in-Depth for Anti- and De-Correlated Random-Dot Stimuli

**DOI:** 10.3390/vision2040041

**Published:** 2018-11-01

**Authors:** Martin Giesel, Alex R. Wade, Marina Bloj, Julie M. Harris

**Affiliations:** 1 School of Psychology and Neuroscience, University of St Andrews, St Andrews KY16 9JP, UK; 2 Department of Psychology, University of York, York YO10 5DD, UK; 3 School of Optometry and Vision Science, University of Bradford, Bradford BD7 1DP, UK

**Keywords:** motion-in-depth, 3D motion, binocular cues, disparity, CD, IOVD, anti-correlation, de-correlation

## Abstract

Motion-in-depth can be detected by using two different types of binocular cues: change of disparity (CD) and inter-ocular velocity differences (IOVD). To investigate the underlying detection mechanisms, stimuli can be constructed that isolate these cues or contain both (FULL cue). Two different methods to isolate the IOVD cue can be employed: anti-correlated (aIOVD) and de-correlated (dIOVD) motion signals. While both types of stimuli have been used in studies investigating the perception of motion-in-depth, for the first time, we explore whether both stimuli isolate the same mechanism and how they differ in their relative efficacy. Here, we set out to directly compare aIOVD and dIOVD sensitivity by measuring motion coherence thresholds. In accordance with previous results by Czuba et al. (2010), we found that motion coherence thresholds were similar for aIOVD and FULL cue stimuli for most participants. Thresholds for dIOVD stimuli, however, differed consistently from thresholds for the two other cues, suggesting that aIOVD and dIOVD stimuli could be driving different visual mechanisms.

## 1. Introduction

Motion-in-depth refers to a movement towards or away from an observer. The detection of motion-in-depth, the discrimination of its direction (i.e., towards or away), and the estimation of its speed are crucial for our survival. For example, judging the speed and direction of a ball coming towards us when playing, e.g., tennis, detecting the deceleration of the car driving in front of us, or predicting whether we will make it across the tracks before being hit by an approaching train, all these tasks require the reliable and accurate perception of motion-in-depth.

When an object moves towards or away from us, the images it projects on the retinas of the two eyes vary systematically with the movement. These variations can be used by the visual system to detect both the direction and speed of motion in depth. Some of these changes can be detected with only one eye (monocular cues). For example, when objects move towards or away from an observer, the size of the retinal images changes (looming): the size increases when the object approaches and decreases when it recedes. Other systematic variations are only detected by comparing the retinal images of the left and the right eye (binocular cues). For example, for a point moving directly towards an observer in depth, the corresponding points in the retinal images move in opposite directions in the two eyes. Both monocular and binocular cues contribute to the perception of motion-in-depth in the real world. However, to study each cue and the mechanisms underlying the processing of the cue separately, stimuli can be created that contain only one type of information. Here, we will be only concerned with the different types of binocular cues to motion-in-depth and will not consider the looming cue.

### 1.1. Types of Binocular Cues to Motion-in-Depth

**Figure 1:**
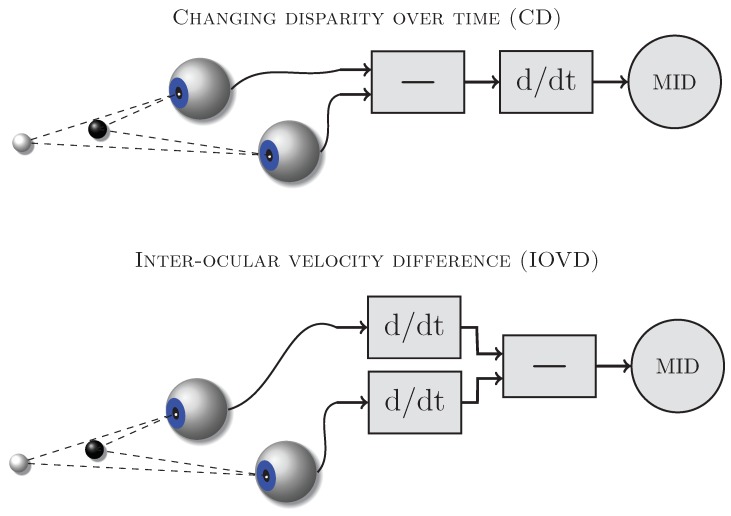
Computational schemes for the CD (**top**) and IOVD (**bottom**) cues (see text for explanation). ‘—’ indicates differencing and ‘d/dt’ differentiation.

Two types of binocular cues might be used by the visual system to detect and discriminate motion-in-depth (e.g., [1,2,3]): changing disparity (CD, Figure 1 top) and inter-ocular velocity differences (IOVD, Figure 1 bottom). Figure 1 schematically shows the computations required to derive these cues from the retinal images. The CD mechanism first computes the disparities between the retinal images in the left and right eye and then determines how those disparities change over time. The IOVD mechanism first computes the velocities of the retinal images separately for the left and the right eye and then compares the two resulting monocular velocity vectors. These cues are mathematically equivalent [3,4] and can provide the same information about moving objects but they differ in the order in which the computations are carried out and therefore potentially require different neural implementations.

### 1.2. Experimental Isolation of the Binocular Cues

**Figure 2:**
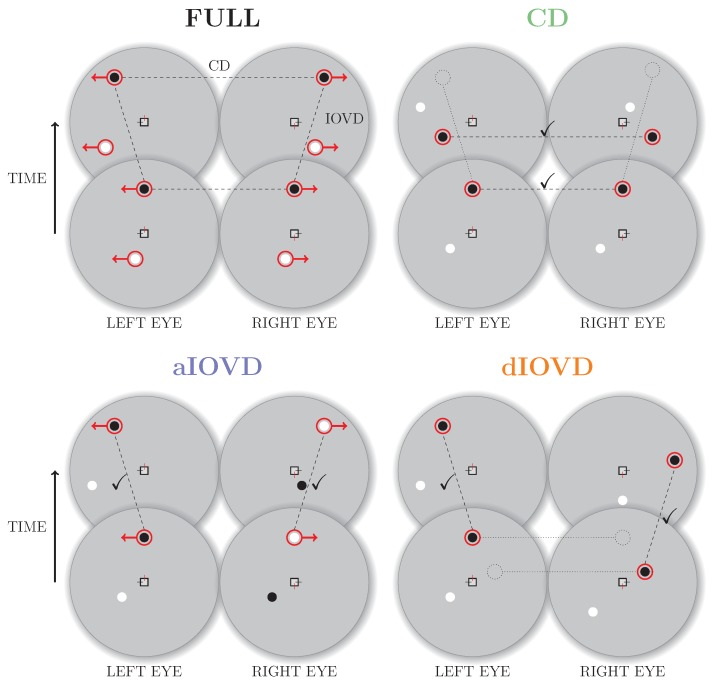
Schematic depiction of two consecutive frames of random-dot stereograms for FULL cue (**top left**), CD (**top right**), aIOVD (**bottom left**), and dIOVD (**bottom right**) stimuli. The grey circles show the stimuli presented to the left and the right eye, respectively, at two consecutive points in time (lower, then upper). Black and white filled dots are examples of random-dots moving on the screen in the direction indicated by the red arrows. Dashed lines connect dots that are correlated between eyes (connecting the left and right eye) and/or correlated between frames (connecting the lower and upper stimulus). Check marks indicate the correlations isolated by the CD and IOVD stimuli, whereas dotted lines and open circles indicate the missing correlations between eyes (dIOVD) and frames (CD), respectively.

Real world motion usually comprises both types of binocular cues. In the following, we will refer to motion-in-depth that combines CD and IOVD information as the FULL cue condition. Using stimuli based on random-dot stereograms it is, however, possible to isolate and selectively probe the CD and IOVD mechanisms [5]. Figure 2 shows a schematic overview of random-dot stereograms combining or isolating the different types of cues.

To create a FULL cue random-dot stereogram, each dot in one eye is paired with a dot of the same contrast in the other eye. The dots move with the same speed in opposite directions creating coherent monocular motion in each eye. Throughout the movement the dots remain at corresponding positions in the two eyes resulting in coherent motion in depth (a change in binocular disparity over time). Note that the dots in the FULL cue stimulus are correlated both spatially (between eyes) and temporally (between frames).

A random-dot stereogram that isolates CD information (also referred to as a dynamic random-dot stereogram) is created by randomly repositioning dots in each video frame so that the changes in binocular disparity remain consistent while the temporal correlations between frames are removed so that there is no coherent monocular motion within each eye’s view. Without consistent monocular motion in each eye, no IOVD cue is available.

A stimulus that isolates IOVD information must generate consistent monocular motion signals in the two eyes without giving rise to coherent changes in disparity. Two methods have primarily been employed to achieve this. The first method is referred to as de-correlated (or uncorrelated) IOVD (dIOVD). It exploits the fact that for the computation of coherent disparity the visual system has to be able to match corresponding elements in the retinal images of the two eyes. This matching process is obstructed or disrupted if the spatial separation between elements in the two eyes becomes too large. In a dIOVD random-dot stereogram dots in one eye have no corresponding dots in the other eye so that the CD cue is minimised. There is, however, consistent dot motion within each of the two eyes (e.g., see [6,7]).

The second method to generate an IOVD isolating stimulus is referred to as anti-correlated IOVD (aIOVD). The aIOVD random-dot stereogram resembles the FULL cue random-dot stereogram with the difference that each dot in one eye is paired with a dot of the opposite contrast in the other eye (inter-ocular contrast reversal), e.g., a black dot in the left eye is paired with a white dot at the corresponding position in the right eye (e.g., see [8]). The rationale for using aIOVD stimuli is that it has been found that perceived depth in static anti-correlated displays is weak or non-existent [5,9,10,11,12].

Objections have been raised regarding the effectiveness of either method to create a stimulus that completely isolates the IOVD cue. Spurious pairings in the dIOVD stimulus might introduce a disparity signal into the stimulus [2,6]. With respect to the aIOVD stimulus, it is unclear whether the lack of static depth perception with anti-correlated stimuli necessarily implies the inability to utilise binocular disparity with this stimulus since V1 neurons sensitive to binocular disparity have been described that respond with an inverted tuning curve to anti-correlated stimuli [13,14,15].

### 1.3. Experimental Evidence for an IOVD-Specific Mechanism

It is still unclear how and where the computations for motion-in-depth are implemented in the brain (for a review see [16]). Existing evidence points to a central role for visual area MT. While it is well established that monkey area MT contains neurons sensitive to motion and disparity (e.g., [17]), evidence for the sensitivity to motion-in-depth is sparse. Several recent studies found evidence for the processing of motion-in-depth in macaque area MT [18,19] and in or around human MT+ [20,21]. Neuronal sensitivity to IOVD stimuli in MT has been demonstrated using de-correlated [19] and anti-correlated [21] random-dot stereograms.

While it has been shown that CD information is sufficient for the reliable perception of motion-in-depth [1,3,22,23], findings for IOVD have been more varied. Some studies found use of the IOVD cue to be absent, or rare [1,24], but others have suggested it is involved in speed discrimination, motion after-effects, adaptation, and the discrimination of the direction of motion-in-depth [7,8,22,25,26,27,28,29,30,31,32,33,34,35,36].

### 1.4. Comparison of aIOVD and dIOVD Stimuli

Only a few perceptual studies on motion-in-depth have used aIOVD stimuli [8,35]. Most other studies have used dIOVD stimuli, but the specific design of these stimuli have varied from standard random-dot stereograms to modified stereograms in which lines of dots alternated with uniform grey bands in counterphase in the two eyes [7] or sparse plaids of drifting Gabors [31]. Shioiri et al. [7] claimed that motion-in-depth only can reliably be discriminated when there is opposing motion in both eyes. One way to achieve this is to present to each eye two random-dot stereograms, one located vertically above the other, where corresponding pairs of dots in the two eyes move in opposite directions, e.g., dots in the upper stereogram move towards the participant while those in the lower stereogram move away, and vice versa. These differences in the stimuli might account for some of the variability in the findings regarding the IOVD mechanism. One converging result seems to be that the IOVD mechanism is more sensitive to higher temporal frequencies and velocities while the CD mechanism prefers lower temporal frequencies and velocities [34,35,37].

To our knowledge no experimental study has so far directly compared aIOVD and dIOVD stimuli. In a modelling study, responses of a motion-energy model [38,39] and a disparity energy model [15] to aIOVD and dIOVD stimuli have been compared [40]. These simulations showed that the direction of motion-in-depth was correctly identified by the motion-energy model for both the dIOVD and the aIOVD stimulus, but the aIOVD stimulus also generated a strong response from the disparity model that was in the direction opposite to the stimulus motion. These computational studies suggest that the different ‘flavours’ of IOVD stimulus might selectively stimulate different mechanisms.

With this in mind, we set out to compare aIOVD and dIOVD stimuli by measuring motion-coherence thresholds for discriminating the direction of motion-in-depth using random-dot stereograms. If one wants to determine the tuning of a neural mechanism, e.g., of the neural units involved in the processing of inter-ocular velocity differences, one has to use stimuli that excite only this particular mechanism. If the stimulus also contained signals that would excite additional neural mechanisms, e.g., neural units that process disparity or looming information, then the resulting data would reflect the properties of some combination of the activated mechanisms. We were particularly interested here in whether we could replicate the similar performances for aIOVD and FULL cue stimuli found previously [35] and to determine whether dIOVD stimuli result in a discrimination performance similar to that found for aIOVD stimuli. We used random-dot stereograms similar to those in a previous study [35], most of our participants were naive, and no feedback was provided during our experiments. We used simulation-based comparisons of different psychometric models to test the hypothesis that the discrimination data for all three motion-in-depth stimuli (FULL, aIOVD, dIOVD) can be fit by a single psychometric model.

Previous studies have almost always used experienced and practiced participants (though see [24] where 60 naive participants were tested). Here, we wanted to include a cohort of naive participants rather than just lab members. For those participants for whom we could determine thresholds for all three cue conditions, we found that discrimination performance for FULL cue and aIOVD stimuli could be described by the same psychometric model for most participants. The novelty of our study was the comparison between responses to aIOVD and dIOVD stimuli, which has not been measured before. Performance for dIOVD stimuli differed, and could not adequately be described by the same psychometric model as FULL cue and aIOVD. This suggests that the detection and discrimination of motion-in-depth for aIOVD and dIOVD stimuli is not mediated by the same single mechanism.

## 2. Methods

### 2.1. Setup

We used a two-monitor mirror stereoscope. The monitors were two CRTs (Iiyama HM204DT A Vision Master Pro 514 22${}^{\prime }$) with a size of 37.5 × 29.5 cm (14.8 × 11.6 inch) and a resolution of 1280 × 1024 pixels and a refresh rate of 85 Hz. The viewing distance was 50 cm. The size of the front silvered mirrors (Edmund Optics) was 7.5 × 7.5 cm. The luminances for black (≈0.02 cd/m${}^{2}$), grey (≈42 cd/m${}^{2}$) and white (≈85 cd/m${}^{2}$) were equated between the two monitors by measuring them from the participants’ viewpoint through the mirrors. The monitors were connected to a PC. The experiment was programmed and run using MATLAB [41] with the Psychophysics Toolbox [42,43,44].

### 2.2. Stimuli

**Figure A1:**
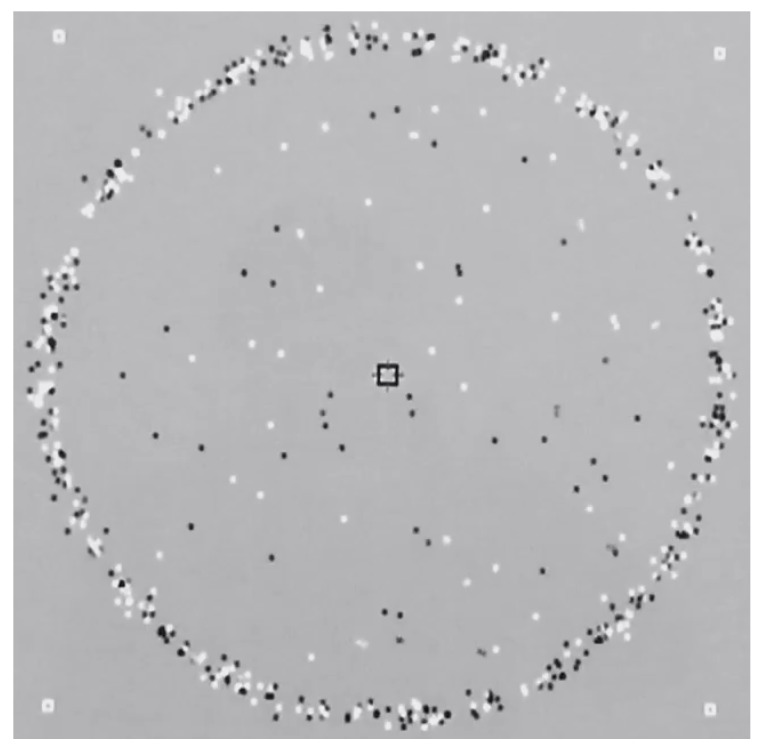
Example of a single frame of the stimulus. In the centre of the left and right eye displays was a square with horizontal and vertical nonius lines of which one line of each orientation was presented to one eye and the other two lines to the other eye. The black and white random-dots moved in a circular field surrounded by a ring of randomly place binocular black and white dots at zero disparity. To help with the binocular alignment of the stimulus white binocular squares were presented in the four corners of the display.

Our stimuli were similar to those used by Czuba et al. [35]. The random-dot stereograms were presented in a circular field with a diameter of 30°. In the centre of the display was a black square subtending 1° with red vertical and black horizontal nonius lines (0.5° length; see Figure A1 in Appendix A for an illustration of the stimulus). The field was surrounded by a ring of static, irregularly spaced black and white dots at zero disparity, on a mid-grey background. Four white squares were placed in the four corners of the screen to help with alignment. We used three types of random-dot stimuli: correlated (FULL), anti-correlated (aIOVD), and de-correlated (dIOVD). For all random-dot stereograms, the monocular fields on the two monitors consisted of 80 black and white dots with a diameter of 0.25°. The dots either belonged to the group of signal dots or to the group of noise dots, and stimuli contained both signal and noise dots in varying proportions (see below).

#### 2.2.1. Signal Dots

For FULL cue and aIOVD stimuli, the change of disparity of the signal dots was consistent with dots traveling through a cylinder in depth towards and away from the participant. Each dot started at a random point in depth and then traversed the volume until it reached one of the cylinder ends at ±0.6° of disparity (from the centre) at which point it ‘wrapped’ (e.g., from the front of the cylinder to the back if it had been moving towards the participant), then continued its trajectory at the opposite end of the volume until it reached its start point. In the case that a dot happened to start at one end of the cylinder, its movement ended at the opposite end. This design deviated from Czuba et al. [35], where each signal dot was assigned the same disparity, with signal dots moving as a plane through depth. The reason for this change was that in the FULL and aIOVD conditions we found the wrapping of the moving plane to be quite conspicuous. It might have tempted participants to respond to the wrap instead of the dot motion. Since the direction of the wrap was always opposite to the direction of the dot movement, it could have been possible for the participants to deduce the correct movement direction from it. Note, that while the motion of the dots in the aIOVD stimulus can be defined by disparity (since there are corresponding dots in the two eyes), the assumption is that due to the interocular contrast reversal this disparity signal cannot be used to perceive motion-in-depth.

The dIOVD stimulus consisted of two clouds of dots: one presented to the left the other presented to the right eye. The two clouds were un-correlated between the eyes, and the dots in each cloud moved into opposite directions. In the dIOVD stimulus dots did not wrap because no dot had a defined disparity since by design there were no corresponding dots in the two eyes. *Monocularly*, dot motion in the three types of stimuli was similar, but they differed in the correlation of the dots between the two eyes. The nominally ‘correct’ direction of motion-in-depth for an IOVD random-dot stereogram is chosen to be consistent with the corresponding FULL cue random-dot stereogram, i.e., if one takes a FULL cue random-dot stereogram whose change in disparity signals motion away from the participant, the dots in each eye move in opposite directions towards the nose. Therefore, an IOVD stimulus with nasally moving dots is consistent with motion away from the participant, while temporally moving dots should signal motion towards the participant.

For all stimuli (FULL, aIOVD, dIOVD), the signal dots travelled with a constant speed of 2.7°/s on the retina. Czuba et al. [35] found that the sensitivity for FULL cue and aIOVD stimuli was higher for faster speeds whereas the sensitivity for CD cues was lowest at high speed. They computed the peak sensitivity for FULL and aIOVD to be around 1.8°/s. We used their highest speed (2.7°/s) to make sure we were working in a range that reduced the sensitivity to CD cues while delivering high sensitivity to IOVD. The signal dots’ life-time, i.e., the number of frames that a dot was visible, was the same as the stimulus duration (19 frames ≈ 224 ms), potentially interrupted by the wrap.

#### 2.2.2. Noise Dots

The motion of the noise dots was a mixture of random re-positioning and a random-walk. This means that some noise dots disappeared after one frame and reappeared at a random position in the next frame (random re-positioning) while others remained ‘alive’ for more than one frame and moved in a randomly determined direction (random-walk). The life-time of the noise dots randomly varied between one and 12 frames following an inverse squared distribution that favours shorter life-times. This mixture of life-times was chosen so that the noise would be equally effective in masking IOVD and CD motion signals [35]. Deviating from Czuba et al. [35], we aimed to deliver noise equivalently in each of the stimulus variants, i.e., the correlational properties of the noise dots differed between the different types of stimuli: The noise dots for FULL cue stimuli were correlated between the eyes, anti-correlated for aIOVD stimuli, and de-correlated for dIOVD stimuli.

### 2.3. Procedure

Before participating in the experiment, the participants’ stereo vision was tested using the TNO test (pass-fail criterion 120 arcsec retinal disparity). Then, participants were instructed that they would see black and white moving dots and that they had to decide whether the dots were moving towards or away from them by pressing one of two keys on a keyboard. They were asked to fixate the fixation marker at the centre of the screen and to try to keep the horizontal and vertical nonius lines aligned. Before beginning the experiment, they were given time to familiarize themselves with the task by doing a test run of the experiment for a few trials. Each participant then received training.

In the training sessions, participants completed 3000 trials in which they were presented with only dIOVD random-dot stereograms at 100% coherence distributed over three sessions on different days. In those trials, they had to decide whether the stimuli moved towards or away from them. Additionally during the training, they were given the option to press a third key to indicate that they were unsure about the direction. We introduced the third response option to get a more nuanced measure of how the participants’ confidence would change during the training. However, participants used this response option very sparingly. No feedback was given. The rationale for the exclusive use of dIOVD stimuli in the training was as follows: the null hypothesis of our study was that performance for the three types of stimuli should be similar. The stimulus properties were chosen to be similar to those used in a study that had found similar performances for aIOVD and FULL cue stimuli [35]. As described above, stimulus properties were optimised for aIOVD but if both aIOVD and dIOVD stimuli isolate the same mechanism their optimal stimulation conditions should be similar. However, it could be that certain aspects in which the stimuli differ result in differences in the optimal stimulus properties. Thus, to mitigate the potential disadvantage of the dIOVD stimulus, we decided to train the participants on dIOVD. So, the training favoured the null hypothesis, i.e., similar performances for all stimuli against which we tested.

Experiments for the three types of stimuli (FULL, aIOVD, and dIOVD) were blocked and their sequence pseudo-randomized. In all experiments, the participants had to decide whether the dots moved towards them or away from them by either pressing the up-arrow key (“away”) or the down-arrow key (“towards”) on a keyboard. Motion coherence, i.e., the ratio of signal dots to noise dots, was varied using the method of constant stimuli. 11 coherence levels were tested ranging from 0% to 100% motion coherence in steps of 10%. The different coherence levels and motion directions were pseudo-randomly interleaved. Participants performed 100 trials at each coherence level (Participant S1 performed 50 trials per coherence level in the FULL condition and 100 trials in the aIOVD and dIOVD conditions). No feedback was given. The measurements were split into two sessions of 50 trials per coherence level for each stimulus type. All measurements were completed in three 1 h sessions on different days. For data analysis, data from the two sessions were combined. The data are available online from the Open Science Framework (https://osf.io/jze5m).

### 2.4. Participants

**Figure A2:**
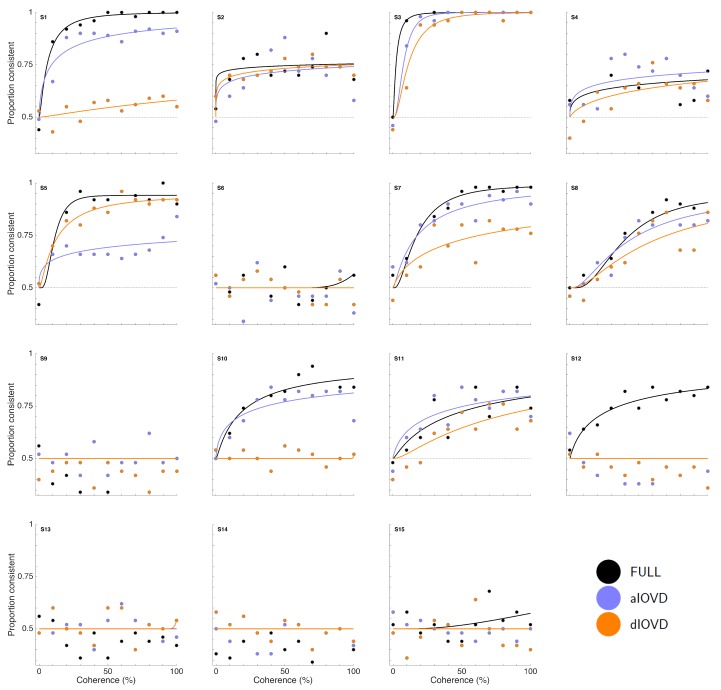
Psychometric function fits for all 15 participants who participated in the screening experiments. The x-axis shows motion coherence as percent signal and the y-axis proportion consistent. Filled circles show data points and curves psychometric functions fit to the data. FULL cue is shown in black, aIOVD in blue, and dIOVD in orange. Note that participant S5 saw motion-in-depth in the direction opposite to the direction that the other participants perceived.

**Figure A3:**
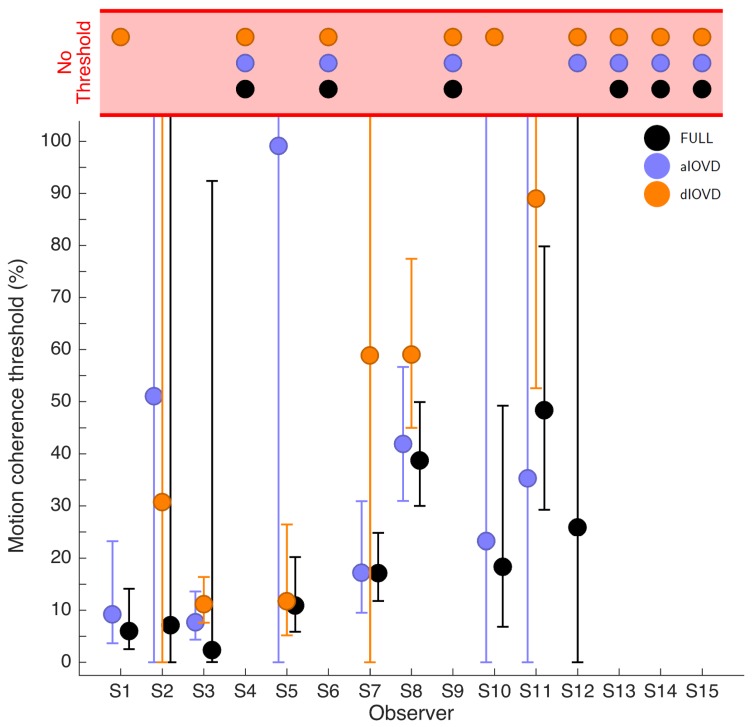
Screening motion coherence thresholds. The x-axis lists the different participants, and the y-axis shows motion coherence thresholds as percent signal. Data for FULL cue are shown in black, aIOVD in blue, and dIOVD in orange. Error bars show 95% confidence intervals of the threshold estimates derived from a non-parametric bootstrap procedure. The horizontal red band indicates for which participants and conditions no thresholds could be determined. Data points have been displaced horizontally to avoid complete occlusion of data points.

We screened a larger number of participants (N = 15, seven female) with a shorter version of the experiment, before the above described training was given. The data from these screening sessions can be found in Appendix B (Figure A2 and Figure A3). Performance was highly variable and frequently very poor. The first six of these participants (three female, chosen by order of recruitment only) went on to complete the training blocks and then the main experiment. Two participants (S1, S2) were lab members and experienced participants in psychophysical experiments, and although not naive as to the purpose of the experiment, they had no prior experience with the specific stimuli used. The other participants were naive volunteers, who were compensated at £5/h for their time. Some of them had prior experience with psychophysical experiments using depth and motion but no exposure to this particular type of experiment or understanding of its purpose. All participants had normal or corrected to normal vision and passed the TNO test. The experimental procedures used were in accordance with the declaration of Helsinki and approved by the St Andrews University Teaching and Research Ethics Committee (Ethics code: PS11472). All participants provided written informed consent before participating in the study.

### 2.5. Data Analysis

**Figure 3:**
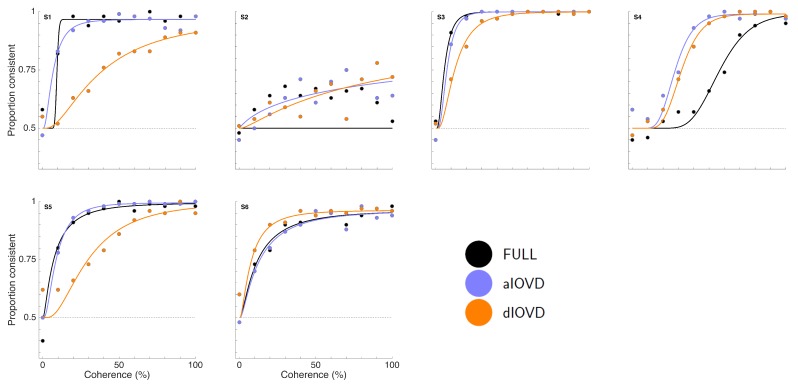
Psychometric function fits for six participants. The x-axis shows motion coherence as percent signal and the y-axis proportion consistent. Filled circles show data points and curves psychometric functions fit to the data. Note that participants S5 and S6 saw motion-in-depth in the direction opposite to the direction that participants S1–S4 perceived.

**Figure 4:**
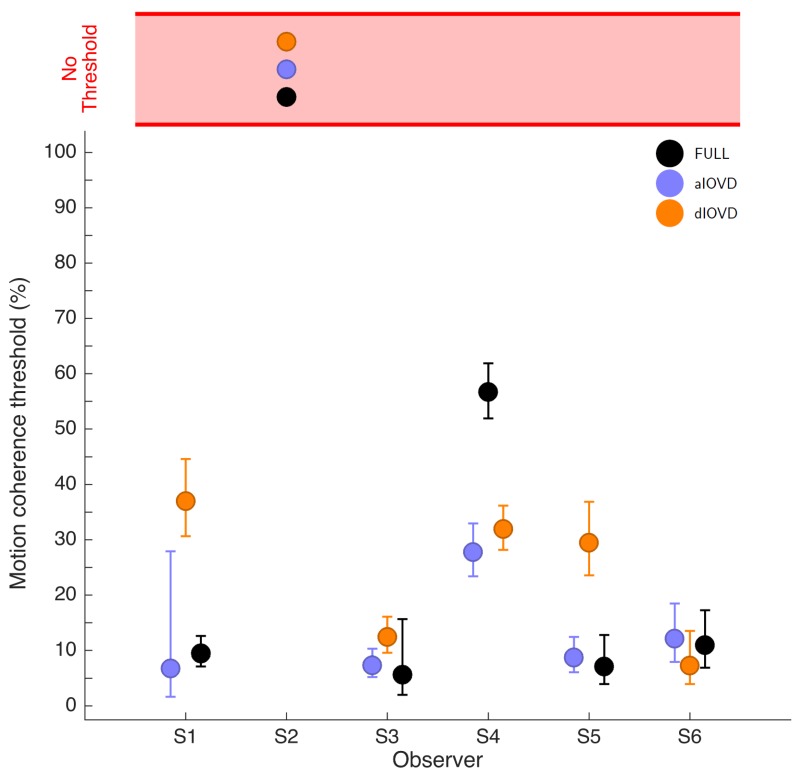
Motion coherence thresholds for the six participants. The x-axis lists the participants, and the y-axis shows motion coherence thresholds as percent signal. Data for FULL cue are shown in black, aIOVD in blue, and dIOVD in orange. Error bars show 95% confidence intervals of the threshold estimates derived from a non-parametric bootstrap procedure. Data points have been displaced horizontally to avoid complete occlusion.

As outlined above we wanted to test whether the same psychophysical model can describe motion-coherence thresholds for all three types of stimuli or whether different models are required to adequately fit the data. To test this, we used the model comparison procedure outlined by Kingdom and Prins [45] in which the data for the different stimulus types are fitted repeatedly under different assumptions. The logic of these model comparisons was as follows: If aIOVD and dIOVD stimuli only contain a velocity and not a disparity signal and hence isolate the IOVD mechanism, and if the performances for FULL cue and aIOVD stimuli are similar as found previously [35], then we would expect to find similar motion-coherence thresholds for all three types of stimuli (FULL cue, aIOVD, dIOVD). In this case, the same psychophysical model should be able to explain the performances for all three types of stimuli. This one-model hypothesis is our null hypothesis. The alternate hypothesis is that not all performances can be explained by the same model because there are differences between performances for some or all stimuli indicating that not all stimuli isolate the same cue to motion-in-depth and that therefore different neural mechanisms are involved in the processing of these stimuli. Our modelling procedure included the following steps:

1. Fitting of psychometric functions
  Cumulative normal psychometric functions were fit to the data using MATLAB^®^ [41] and the Palamedes toolbox [46]. Initially, we fitted psychometric functions separately for each participant and condition with fixed guess rate (0.5) and fixed lapse rate (0.01). The resulting threshold and slope parameter estimates were then used as starting values for fitting data from the three stimulus conditions (FULL, aIOVD, dIOVD) simultaneously for each participant. In these fits, the lapse rate parameter was free to vary between participants but not between conditions to estimate a single lapse rate for each participant for all conditions. The range of possible lapse rates was constrained to values between 0 and 0.06. The fits are shown in Figure 3.
  The errors associated with the parameters determined by fitting psychometric functions (thresholds, slopes, and lapse rates), were estimated by performing 2000 non-parametric bootstraps of the fits. All simulations converged. The standard error (*SE*) of the parameter estimates is given by the standard deviation of the sampling distribution of parameter estimates. We present 95% confidence intervals representing ±1.96 *SE*.
  Motion coherence values ranged from 0–100% in steps of 10%. These values were log-transformed before fitting the cumulative normal function. For clarity, the thresholds and corresponding confidence intervals are displayed on a linear scale in Figure 3 and Figure 4. The transformation from log to linear values resulted in asymmetric error bars.
2. Model comparison
  Our aim was to determine whether the three stimulus conditions affected performance differently. To do this, we compared two different models:
  **Model 1**: we assumed that the stimulus conditions did not affect performance differently, i.e., all potential differences between the conditions would be due to sampling, while the underlying thresholds and slopes would be the same in all conditions. In this case, the same psychometric function would adequately fit data from all conditions.
  **Model 2**: we made the assumption that the different conditions affect performance in different ways. In this case, separate psychometric functions would have to be fit to the data indicating that the performance is not determined by the same single underlying mechanism.
  To determine which model provided the better fit, data from all three conditions were fit twice: once under the assumptions of each of the two models. For fitting Model 1, the data from all conditions were combined, and for Model 2, the conditions were fit separately. Then the likelihood ratio between the first and second model fits was computed. The second model has more free parameters than the first model. So, the first model can never provide a better fit than the second model. A likelihood ratio of one would indicate that the two models fit the data equally well. The smaller the likelihood ratio, the worse is the fit of the first model relative to the second model. Note that the model comparison compares the fits of the two models. It does not check whether the models themselves provide a good fit to the data. This is done by the goodness-of-fit test.
  The single likelihood ratio between the two models alone does not allow us to say whether the data can be sufficiently explained by the first model or not because the differences could be due to sampling. The appropriate question to ask is: assuming that the data can be described by a single model, how likely is it that we find a likelihood ratio between the two models as low or lower than the one that we found for the experimental data?
  To determine whether the likelihood ratio could be explained by sampling alone, a ‘simulated participant’ was created who responded according to the first model, i.e., random data sets were repeatedly generated based on the psychometric function fitted to the combined experimental data. The two models are fitted to the simulated participant data and for every simulation, the likelihood ratio between the two models is calculated. In this case, we know that the first model must provide a good fit to the data and that all fits resulting in a likelihood ratio smaller than one are due to sampling. The likelihood ratio for our simulated data sets is then compared to the likelihood ratio between the two models that was found for the fit to the experimental data. The proportion of simulations (*p*) that resulted in a likelihood ratio smaller than the likelihood ratio for the experimental data indicated whether the experimental likelihood ratio was in the range of the likelihood ratios expected due to sampling.
  We then set a value for *p* below which we assumed that it to be unlikely that a participant who behaved according to the first model would produce likelihood ratios as small or smaller than those found for the experimental data. In this case, we rejected the null hypothesis that the stimulus conditions did not affect performance differently and instead assumed that different psychometric functions are required to adequately describe the data.
  We chose a cut-off value of α=0.05 for *p* and used 2000 bootstraps for each model comparison and participant. All simulations converged.
3. Goodness-of-fit
  A goodness-of-fit analysis was used to test the assumptions made during the fitting procedure. We assumed that the psychometric functions were cumulative normal functions with a guess rate of 0.5 and lapse rates between 0 and 0.06 that were equal between conditions. These assumptions specified the target model which was then tested against a model that made no specific assumptions (saturated model), i.e., that was based on the observed proportions of correct responses alone. Both models were fit to the experimental data and the likelihood ratio of the fits was computed. The same test was performed repeatedly with simulated data generated based on the target model. For each simulated data set, the likelihood ratio for the fit of the target model to the simulated data and the fit of the saturated model were computed. The proportion of simulations (*p*) that resulted in a likelihood ratio smaller than the likelihood for the experimental data indicates whether the target model provides a good fit to the experimental data (see [45]). We assumed that if this goodness-of-fit measure *p* was smaller than 0.05 the fit was unacceptably poor (as per [45]), then the target model did not represent a good fit to the data. The experiment was simulated 2000 times, and all simulations converged. The results of the goodness-of-fit test are shown in Figure A6 in Appendix C.

## 3. Results

Figure 3 shows proportion consistent versus percent motion coherence for the six participants for FULL cue (black) aIOVD (blue) and dIOVD (orange). Solid lines show fitted cumulative-normal psychometric functions. By ‘correct’, we would normally refer to the direction of motion specified by the IOVD signal (see above). While the changes in disparity in random-dot stereograms might be equivalent to those found in real-world motion-in-depth, the full-field IOVD signal, because looming cues have been removed, generates a set of motion-in-depth vectors that would be consistent with complex non-rigid motion in the real world [16]. The entire stimulus, with all its cues, is therefore technically consistent with a number of different motion interpretations. We found that each participant was consistent in their own interpretation of direction (and were therefore able to achieve a threshold) but the polarity of the interpretation was not constant from participant to participant. For participants S1–S4, the interpretation was consistent with the direction of IOVD. For S5 and S6 it was consistent with the opposite direction. We gave no feedback here, and thus such differences in interpretation are not unexpected (see also [47,48,49]). To be able to fit psychometric functions to all data and compare performances of participants, we chose for each participant the response coding with the highest consistency with their responses and determined proportion correct with respect to this coding of the responses. We refer to this measure as ‘proportion consistent’. Participants S1–S4 shared the same coding, while the coding for S5 and S6 was reversed.

Figure 4 shows the 75% motion-coherence thresholds. The horizontal red band indicates conditions where no threshold could be obtained.

**Figure A4:**
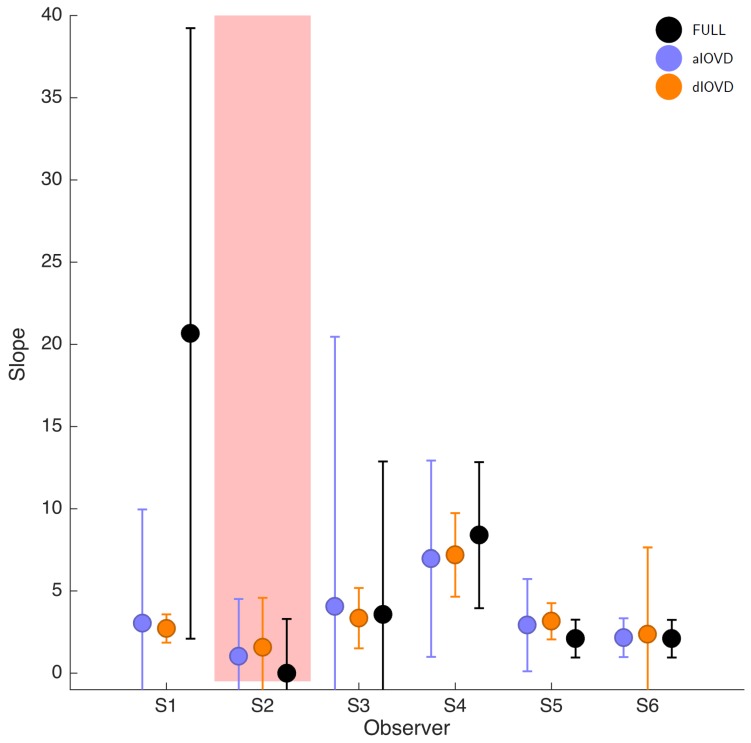
Slopes (in log-space) for six participants. FULL cue is shown in black, aIOVD in blue, and dIOVD in orange. Error bars show 95% confidence intervals of the slope estimates derived from a non-parametric bootstrap procedure. The red shaded area indicates participants for whom no thresholds could be determined.

**Figure A5:**
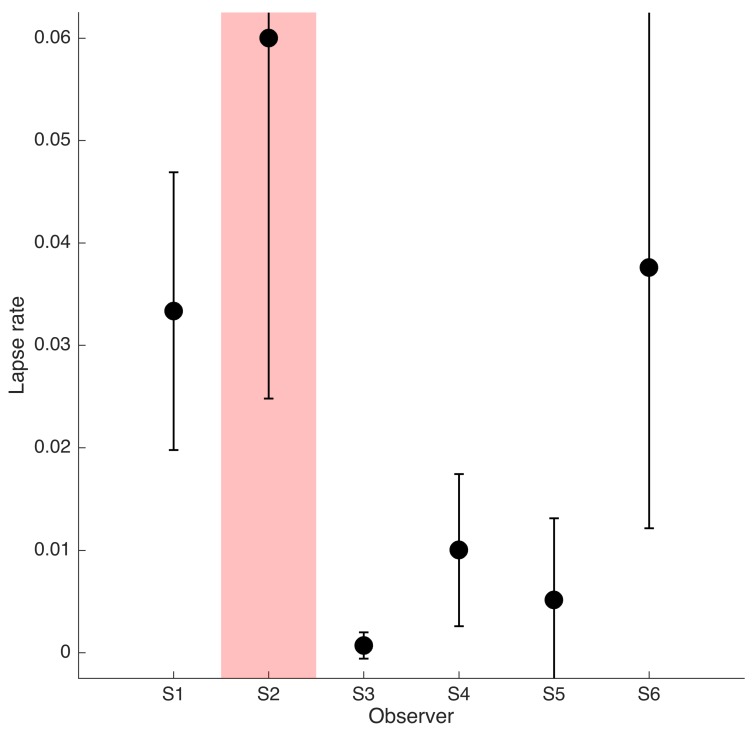
Lapse rates for six participants. Lapse rate fits were constraint to be identical for the three stimulus types and limited to the range 0–0.06. Error bars show 95% confidence intervals of the lapse rate estimates derived from a non-parametric bootstrap procedure. The red shaded area indicates participants for whom no thresholds could be determined.

**Figure A6:**
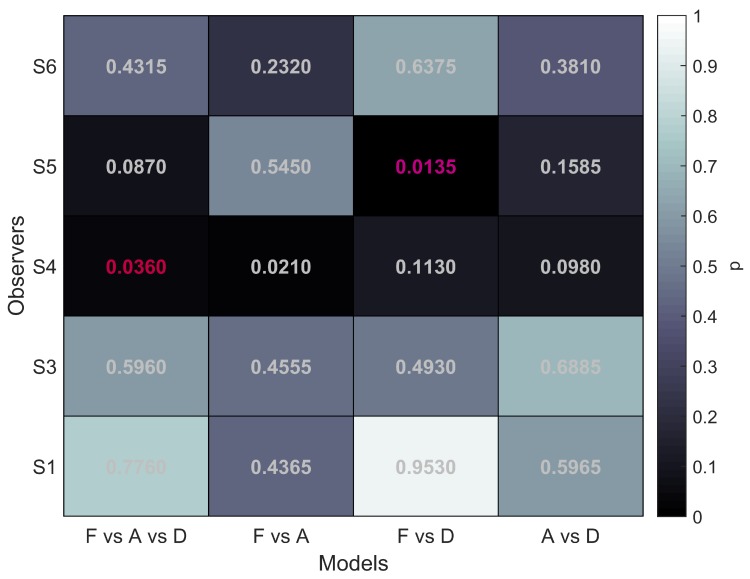
Goodness-of-fit for four models. The different models are shown on the x-axis: F vs. A vs. D, F vs. A, F vs. D, A vs. D with F: FULL cue, A: aIOVD, and D: dIOVD. The y-axis shows the five participants that were included in the analysis. Grey-shading and values in the different fields indicate the *p*-values for each test. The significance level for the overall comparison (first column) was α=0.05 (significant values are shown in red). For the multiple comparisons (columns 2–4) it was adjusted to $\alpha_{\mathrm{bc}}=0.0167$ (significant values are shown in magenta).

For participant S2, no thresholds could be determined due to poor performance, even for the highest coherence levels (Figure 3). This participant was excluded from the subsequent analysis. For the other five participants we could determine thresholds for all three types of stimuli. Excluding participant S2, for four of five participants, the thresholds for FULL cue and aIOVD stimuli were similar. For three of these participants, thresholds for the dIOVD stimulus were higher than those for FULL and aIOVD stimuli. For one participant (S6), the threshold for dIOVD was lower. Participant S4 differed from the other participants in that they had similar thresholds for aIOVD and dIOVD stimuli that were clearly lower than the threshold for the FULL cue stimulus. The psychometric functions for this participant also exhibited a different shape compared to those of the other participants (Figure 3). The psychometric function slopes for the three stimulus types were similar to each other for most participants (see Figure A4 in Appendix C). As described in the Methods section, one lapse rate was fitted for each participant for all conditions. The lapse rate was allowed to vary between 0 and 0.06. Figure A5 in Appendix C shows that lapse rates were well below 0.06 for all participants except for the excluded participant S2.

The differences between thresholds that we found—especially between aIOVD and dIOVD stimuli—might indicate support for different mechanisms underlying the detection of motion-in-depth for aIOVD and dIOVD stimuli. To analyse this in more detail we used model comparisons.

### Model Comparison

To evaluate the performance differences between the different stimulus types we performed model comparisons following the recommendations by [45]. For these comparisons the data sets of participants S1, S3, S4, S5, and S6 were used. The analysis was performed separately for each participant. Details are described above in Methods. First, we determined whether there was an overall difference between the function fits for the three stimulus conditions. We refer to this as the F vs. A vs. D comparison with F referring to FULL, A to aIOVD, D to dIOVD. Additionally, we performed multiple pairwise comparisons testing models F vs. A, F vs. D, and A vs. D. This procedure is akin to performing a one-way ANOVA with stimulus condition as factor followed by multiple pairwise comparisons.

The null hypothesis for each comparison was always that the performance for the conditions that are compared can be fit by the same psychometric function, indicating that a single mechanism might underlie the detection and discrimination of motion-in-depth for the stimuli that were compared. Based on the results by Czuba et al. [35] we would expect that in the F vs. A test the null hypothesis would not be rejected.

**Figure 5:**
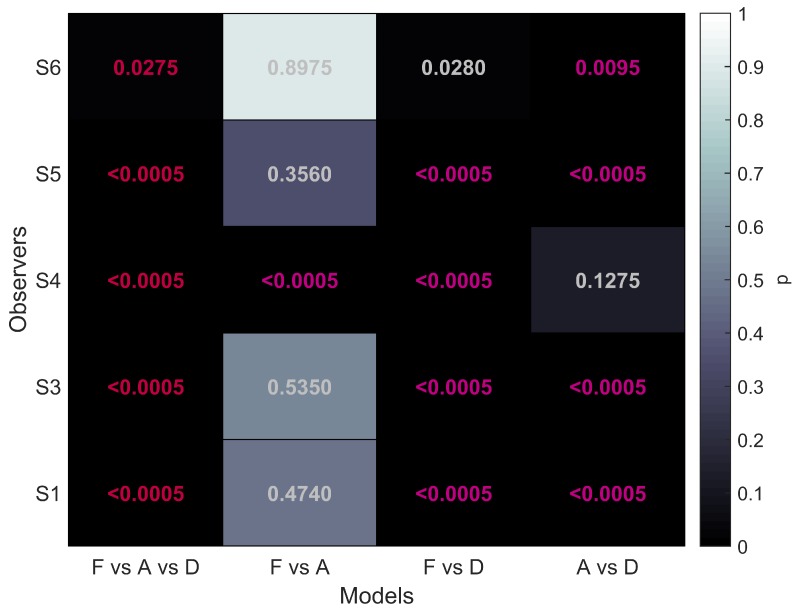
Model comparisons for four models. The different models are shown on the x-axis: F vs. A vs. D, F vs. A, F vs. D, A vs. D with F: FULL cue, A: aIOVD, and D: dIOVD. The y-axis shows the five participants that were included in the analysis. Grey-shading and values in the fields indicate the *p*-values for each comparison. The significance level for the overall comparison (first column) was α=0.05 (significant values are shown in red). For the multiple comparisons (columns 2–4), it was adjusted to $\alpha_{\mathrm{bc}}=0.0167$ (significant values are shown in magenta).

Since psychometric function fits could vary between conditions in both thresholds and slopes, differences in performance can result in differences in thresholds and/or differences in the slope and we thus looked at both. The guess rate was fixed, and the lapse rate varied only between participants but not between conditions. The significance level for the overall comparison (F vs. A vs. D) was α=0.05. For the multiple comparisons the (conservative) Bonferroni corrected value $\alpha_{\mathrm{bc}}=0.05/3=0.0167$ was used. The results of the model comparisons are shown in Figure 5.

The first column of Figure 5 shows that for all five participants the overall comparison of FULL cue, aIOVD, and dIOVD stimuli resulted in significant *p*-values. This means that for all participants the null hypothesis that the same psychophysical model can describe performances for all stimuli was rejected. At least one stimulus type resulted in different discrimination performances.

The second column of Figure 5 represents the comparison of FULL cue and aIOVD stimuli. For four out of five participants the null hypothesis could not be rejected, i.e., indicating that as expected from previous results data from FULL cue and aIOVD stimuli can be described by the same model. Therefore, performances for those stimuli were likely based on the same motion-in-depth mechanisms.

The comparison between FULL cue and dIOVD stimuli (third column), indicates that performance for these two stimuli differed significantly for four out of five participants. A similar significant difference for four of five participants was found for the comparison of aIOVD and dIOVD stimuli (fourth column).

For most of the participants (S4 being the outlier), performances for aIOVD and dIOVD stimuli cannot be described by the same psychophysical model. This suggests that different visual cues were used for the detection of motion-in-depth for aIOVD and dIOVD stimuli. This contradicts the assumption that aIOVD and dIOVD stimuli isolate the same IOVD mechanism.

## 4. Discussion

We compared performances for the discrimination of the direction of motion-in-depth for three types of random-dot stimuli (FULL cue, aIOVD, dIOVD) using motion coherence thresholds. aIOVD and dIOVD stimuli are designed to only contain velocity cues to motion-in-depth but no reliable disparity signal. Our main interest was to test whether this assumption is true. We wanted to determine whether discrimination performances for both aIOVD and dIOVD random-dot stereograms actually rely on the same type of cue to motion-in-depth, i.e., inter-ocular velocity differences, and are therefore processed by the same neural mechanism (IOVD mechanism).

### 4.1. Comparability of aIOVD and dIOVD Stimuli

For most participants, we found similar performances for FULL cue and aIOVD stimuli. We showed that the same psychophysical model provided a good fit to the discrimination data for both FULL cue and aIOVD stimuli. This is in accordance with the results by Czuba et al. [35] and suggests the same underlying mechanism may be being used to discriminate the motion direction. Performance for the dIOVD stimulus, however, differed from those for the aIOVD stimulus for all but one participant. A different psychophysical model would be required to fit the data for the dIOVD stimulus compared to the FULL cue and aIOVD stimuli. Thus, we suggest that these data suggest that the detection of motion-in-depth for aIOVD and dIOVD stimuli is based on different cues to motion-in-depth.

Why would performances between aIOVD and dIOVD stimuli that are supposed to isolate the same cue to motion-in-depth differ? Our stimuli were chosen based on the results by Czuba et al. [35] who found with their stimuli similar motion-coherence thresholds for FULL cue and aIOVD stimuli. The general, yet so far untested, assumption has been that both aIOVD and dIOVD stimuli isolate the IOVD mechanism by either rendering disparity information unusable (aIOVD) or by removing it (dIOVD) so that the detection and discrimination of motion-in-depth can only rely on the velocity information in the two eyes. No matter the method (aIOVD or dIOVD), this remaining velocity information should be very similar for the two types of IOVD stimuli since monocularly aIOVD and dIOVD stimuli were similar, i.e., the dots had the same size, contrast and, most importantly, the same monocular speed in the two eyes. The stimuli did also not differ systematically in other monocular cues (looming, optic flow) or in extra-retinal cues. Therefore, detection performances for aIOVD and dIOVD stimuli should be similar and should reflect the sensitivity of the IOVD mechanism.

Different performances for aIOVD and dIOVD stimuli could indicate that performance is not based on the same mechanism. Either one of the stimuli, or both, could insufficiently isolate the IOVD mechanism. The main concern is that differences in performances between the IOVD stimuli might be the result of a contamination of the velocity signal by a maybe weak but still consistent disparity signal in the stimulus. Then there are two possible explanations for the similar performances for aIOVD and FULL stimuli found here and previously [35]. The first possibility is that velocity information is the dominant binocular cue for the detection and discrimination of motion-in-depth and therefore determines the performance for the FULL cue stimulus which contains both disparity and velocity information. In this case, one would expect the performance for dIOVD stimuli to be similar to those for FULL cue and aIOVD stimuli. The second possibility is that the visual system is able to extract consistent disparity signals from anti-correlated random-stereograms moving in depth and that, therefore, the aIOVD stimulus contains, similar to the FULL cue stimulus, both disparity and velocity information resulting in similar detection performances for those two stimuli. In this case, one would expect, assuming that the dIOVD stimulus does not contain a consistent disparity signal which by design it should not, that performances for the dIOVD stimulus should be different from those for FULL cue and aIOVD stimuli. Our results favour the second explanation.

### 4.2. Inter-Individual Variability

Especially in the screening data, we found wide inter-individual variability both in the general ability to perceive the direction of motion-in-depth with random-dot stereograms and in the preferences for different types of stimuli for motion-in-depth. Given that most previous studies investigating the perception of motion-in-depth used relatively small sets of participants, who in many cases were the authors themselves and/or highly experienced, the variability in performance between participants and a widespread inability of being able to perceive motion-in-depth in random-dot stereograms might often have gone unnoticed. This is corroborated by findings from a recent study that systematically investigated the effectiveness of static and dynamic stereoscopic stimuli for a sample of 127 participants [50]. Using naive participants, and no feedback, as we did here, aims to provide a realistic picture of the ability to perceive motion-in-depth with random-dot stereograms. Given the artificial and impoverished nature of random-dot stimuli compared to real-world motion-in-depth, participants might resort to using various strategies when forced to make a decision about the direction of motion-in-depth.

Wide variation in the perception of motion-in-depth from large numbers of participants using random-dot stereograms has been reported previously by Nefs et al. [24]. They also noted that their experienced participants were not necessarily better than naive participants. We found a similar pattern in the screening data (Figure A2 and Figure A3). Naive participant S3 performed better than the experienced participants S1 and S2. Participant S1 had previous experience with a different type of dIOVD random-dot stereogram and could detect motion-in-depth with that stimulus, but at first still could not perceive it with our dIOVD stimulus. This could indicate that performance might be very stimulus-specific. Training as provided in our experiment, i.e., without feedback, seemed to have improved performance for some participants but not for all, e.g., participant S2. Although training was only done with dIOVD stimuli, improvements due to training, when present, seem to have generalized to the other stimulus types.

Czuba et al. [35] did not present individual data from their three experienced participants and most of our participants’ thresholds (ignoring the variations in the perceived direction of motion-in-depth) for FULL cue and aIOVD stimuli are in the same range as for the group data they presented. This suggests that the subtle differences in stimulus designs did not have a large effect on performance.

One interesting aspect of the variability between participants that we found was that two participants responded as seeing motion-in-depth in the opposite direction to that specified by the IOVD signal. Apart from this their performances did not differ systematically from those of the other participants. As has been noted many times before (e.g., [16,51,52]) there are many cues to motion-in-depth, and the failure to correctly detect and discriminate motion-in-depth in random-dot stereograms may not be indicative of a general inability to perceive real world 3D motion. It might therefore be that, for individuals such as S5 and S6, extraretinal or looming cues are required for veridical perception of motion-in-depth in everyday situations [48,51]. For example, participants S5 and S6 could have relied on an optic-flow signal. In random-dot stereograms, dots that move temporally (i.e., to the left in the left eye view and to the right in the right eye view), deliver binocular information that signals motion away. However, the temporal movement of the dots would also be consistent with the monocular looming cue that signals an observer moving towards the screen (and vice versa for nasally moving dots). If observers based their decisions on a monocular subset of dots at the stimulus boundary, they could respond in the direction opposite to the binocular cue. In general, the information content of real world motion-in-depth is much richer than the motion-in-depth simulated by random-dot stereograms moving on a screen.

An alternative explanation for the reversal is as an artefact of our stimulus design consistent with participants basing their decisions on the wrapping of the dots to the opposing end of the stimulus trajectory. While this is at least theoretically possible for the FULL cue and aIOVD stimuli, it cannot explain the inversion for the dIOVD stimulus because of the absence of a wrap in this stimulus.

One of our participants (S4) exhibited a distinctly different pattern of thresholds with better performances for aIOVD and dIOVD than for FULL cue stimuli. Nefs et al. [24] identified a subgroup of participants that seemed to prefer IOVD cues over CD cues. Participant S4 could belong to this group. The model comparisons showed that for this participant—in contrast to all other participants—the same model can describe the performances for aIOVD and dIOVD stimuli but not for the FULL cue stimulus. This would be consistent with a low sensitivity to the disparity signal compared to sensitivity to the velocity signal. But we cannot explain why poor sensitivity to the CD cue in the FULL stimulus would result in poorer performance, unless some highly non-optimal form of cue combination was at work.

## 5. Conclusions

The aim of this study was to make a direct comparison of aIOVD and dIOVD stimuli for driving the perception of motion-in-depth. We have shown that performance is systematically different for the two cues, and thus our data suggest that they may drive different mechanisms for motion-in-depth perception.

## Appendix A. Additional Methods

Figure A1 shows an example of the stimulus.

## Appendix B. Screening Data

### Appendix B.1. Methods

15 participants (seven females) completed the screening measurements. Two further participants started the experiment but did not complete it. Their data were excluded. Two participants (S1, S2) were lab members and experienced participants in psychophysical experiments, and not naive as to the purpose of the experiment but had no prior experience with the specific stimuli. 13 participants were naive volunteers, who were compensated at £5/h for their time. Some of them had prior experience with psychophysical experiments using depth and motion but no exposure to this particular type of experiment. All participants had normal or corrected to normal vision and passed the TNO test. The experimental procedures used were in accordance with the declaration of Helsinki and approved by the St Andrews University Teaching and Research Ethics Committee (Ethics code: PS11472). Of these 15 participants, six participants (S1–S6) participated in the main experiment. All participants provided written informed consent before participating in the experiment.

In the screening sessions, participants completed 50 trials at each of the 11 coherence levels (Participant S1 performed 50 trials per coherence level in the FULL condition and 100 trials in the aIOVD and dIOVD conditions.). The measurements were completed in two 1 h sessions on different days.

### Appendix B.2. Results

Overall, we found that performance for all binocular cues to motion-in-depth varied widely between participants, with very few being able to see motion-in-depth from all cues before training. Specifically, we found that many of our participants could not reliably perceive motion-in-depth for some or all stimulus types, but there was no overall clear pattern.

The psychometric functions for 15 participants are shown in Figure A2. First, we note that for some conditions, participants’ data were too poor to obtain a reliable threshold fit. For example, for participants S9 and S13–S14, performance does not improve as the proportion of signal dots is increased, as would be expected if participants were sensitive to motion-in-depth. Such datasets are indicated by a point in the reddish upper-band in Figure A3, which summarises screening threshold fits (75% thresholds) where they could be measured.

One participant (S5) consistently perceived motion-in-depth in the direction opposite to the direction perceived by the other participants. The participant was not made aware of this reversal but repeatedly reminded of the correct assignment of the response keys to motion directions. For this participant, the coding of the responses was reversed compared to the coding for the other participants.

For six of 15 participants we could determine thresholds for all three conditions, but confidence intervals for the threshold estimates were often large. Looking again at the psychometric functions in Figure A2, only participant S3 showed consistently reliable performance for all three cue conditions. For six participants, no thresholds could be determined for any of the three stimulus conditions. Based on the number of undetermined thresholds, dIOVD (9/15) was the most difficult condition, followed by aIOVD (7/15), and FULL (6/15). Most participants informally reported that they found the task difficult and did not see clear motion-in-depth, but instead saw a variety of types of motion, e.g., rotations, expansions, contractions, or lateral motion. Six of the 15 participants (S1–S6) participated in the main experiment.

## Appendix C. Additional Results

Figure A4 shows the psychometric function fit slopes, for the functions displayed in Figure 3.

Figure A5 shows the lapse rates.

Figure A6 shows the results of the goodness-of-fit test. The null hypothesize for the goodness-of-fit test is that the chosen psychometric model is adequately fitting the data. Significant *p*-values indicate that the chosen model does not provide a good fit. The significance level for the overall test (first column) was α=0.05 and $\alpha_{\mathrm{bc}}=0.0167$ for the multiple comparisons (second to fourth column).

The tests for participants S1, S3, S6 were clearly not significant, indicating an acceptable fit of the psychometric model. The goodness-of-fit was lower for participants S4 and S5 but only two comparisons resulted in significant *p*-values.
